# Microscopic Tubulovenous Communications in Nonneoplastic Kidneys: A Single-Center Case Series and Review of the Literature

**DOI:** 10.1016/j.xkme.2025.101071

**Published:** 2025-07-16

**Authors:** Cullen M. Lilley, Jonathan E. Zuckerman

**Affiliations:** UCLA Health, Department of Pathology and Laboratory Medicine, Los Angeles, CA

**Keywords:** Hematuria, kidney injury, nephropathology, Tamm-Horfall polyps, tubulointerstitial disease, tubulovenous fistula, tubulovenous herniation

## Abstract

**Rationale & Objective:**

Pathological connection between the kidney tubules and veins is known as a microscopic tubulovenous communication we refer to as a tubulovenous fistula (TVF). This finding has been reported in a few small case reports, but no systematic examination of cases across various clinical settings detailing their histologic spectrum and associated clinical/pathologic findings has been performed.

**Study Design:**

Case series and literature review.

**Setting & Participants:**

Nonneoplastic kidney pathology reports from an academic medical center (February 1, 1990, to February 1, 2024) were queried for mention of TVF. In total, 30,537 nonneoplastic kidney reports were queried, and 22 cases of TVF were identified.

**Results:**

In total, 72.7% of TVF cases were from native kidney biopsies. Median patient age was 66 (range, 25-84) with a male predominance (68.2%). Clinically, 82.4% had microscopic hematuria, and 17.6% had gross hematuria. TVFs were usually singular and involved arcuate size veins. Microscopically, 95.5% of cases had acute tubular injury, and 73.3% had at least focal pathologic intratubular casts/calcium crystals. In total, 56.3% of native cases had interstitial nephritis. Of the transplant cases (n = 6), 66.7% exhibited rejection.

**Limitations:**

Laboratory data, clinical follow-up, and original slides were not available in all cases examined. Although rare in our repository, TVFs are likely an under-reported finding. In addition, the focality of TVFs could play a role in their limited rate of detection.

**Conclusions:**

This is the largest case series exploring the clinical and histologic features associated with TVFs in the kidney. Our findings support the assertion that TVFs are associated with hematuria without glomerulonephritis and occur in the setting of significant tubular injury, intratubular casts/crystals, and obstructive phenomena likely because of disruption of tubular basement membranes adjacent to veins.

## Plain Language Summary

Our study explores a rare feature found in medical kidney biopsies called tubulovenous communications, in which small blood vessels connect with kidney tubules. These communications can sometimes be overlooked or not recognized because of their rarity. We reviewed a series of biopsies to identify and describe these communications and their associated findings in the kidney. By looking at these biopsies, we learned that these communications are more common when there is background kidney inflammation. Our findings highlight the importance of recognizing tubulovenous communications, which could potentially explain blood in the urine in patients with kidney inflammation but no damage to the glomerulus. Our findings also suggest that inflammation may be a mechanistic explanation for the formation of these connections.

A pathologic connection between the vasculature and tubules is a rare histologic finding observed on examination of kidney biopsy sections. This pathological tubulovenous communication has been referred to as a tubulovenous herniation, Tamm-Horsfall polyp, or a tubulovenous fistula (TVF). Because the tubulovenous communication seen in these cases forms a luminal connection between these 2 structures, we refer to this finding as a TVF. Although the pathogenesis and clinical significance of this finding have yet to be established, prior case reports have described this finding in association with crush injuries,^1^ vesicoureteral reflux,^2^^,^^3^ interstitial nephritis,^4^ acute tubular necrosis,^3^^,^^5^ allograft rejection,^4^^,^^6^ multiple myeloma,^7^ medical instrumentation,^8^ or with no apparent concurrent pathology.^9^, ^10^, ^11^ The detection of a TVF in a kidney biopsy also provides a potential explanation for hematuria in cases in which glomerular etiologies have been excluded.

**Figure 1:**
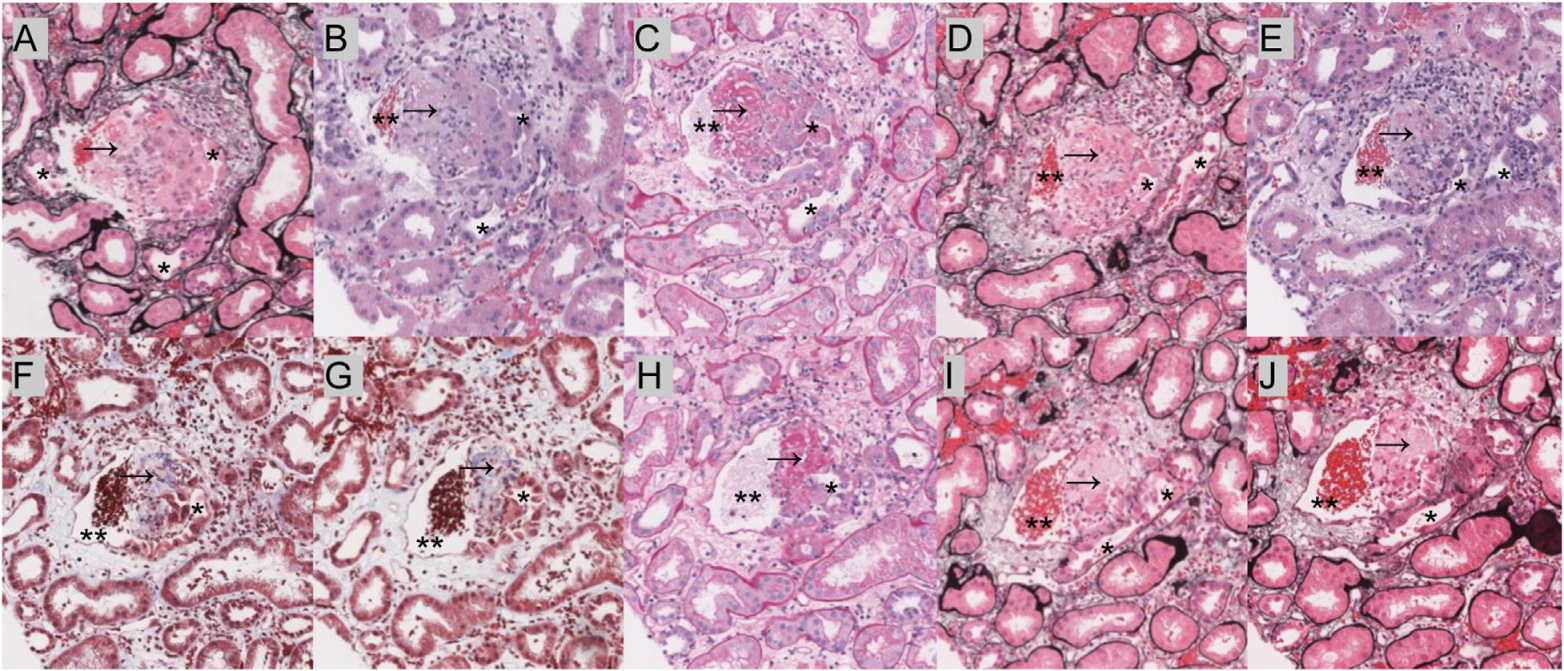
Evolution of a tubulovenous fistulas over serial histologic sections. Serial histologic photomicrographs depicting the demonstrated connection between the tubule (∗) and vein (∗∗) with Tamm-Horsfall protein (arrow) in the venous lumen. Histologic sections are arranged in order of serial sectioning. Sections stained with Jones’ silver (A, D, I, J), hematoxylin and eosin (B, E), periodic acid-Schiff (C, H), and trichrome (F, G) stains.

Histologically, a TVF is defined as a focal connection between a vein and a tubule apparent on light microscopy based on the characteristic identification of Tamm-Horsfall protein (THP) (also known as uromodulin) within the lumina of the vein. In such cases, THP can be present in venous lumina in isolation, admixed with fibrin, or in association with mixed inflammatory cells and/or tubular epithelial cells (Fig 1). As such, the identification of abnormal localization of THP can be crucial in identifying potential underlying pathologic states or serve as a clue to search for a nearby TVF.

Aggregates of THP are readily identifiable on histologic sections by their waxy, eosinophilic appearance on hematoxylin and eosin, bright periodic acid-Schiff positivity, negativity by Jones’ silver, and pale blue coloration on trichrome staining.^12^^,^^13^ In indeterminate cases, immunostaining for THP/uromodulin or ultrastructural examination can be performed; however, few clinical nephropathology laboratories employ this stain. Because THP is secreted into the tubular lumen by cells in the thick ascending limb of the loop of Henle and excreted into the urine, collections of THP in other sites can point to a pathologic state. For instance, in reflux states, THP can be displaced into the thin limbs of the loop of Henle, the proximal tubules, or even Bowmen’s capsule.^14^ In states of tubular rupture, THP can be identified in the in the interstitium.^15^

Although a TVF is a rare entity and THP is typically readily identifiable on histologic examination, there are several factors that affect its detection and identification. Diagnosing TVF on histologic examination is partial clouded by a lack of awareness of the entity, but the diagnosis also relies on the examination of multiple histologic levels to determine the presence of the, often small and focal, pathologic connection between the tubule and vein. On initial examination, the range of histopathologic presentations should be taken into account to determine if further work-up or deeper sections need to be obtained. Currently, however, there is a paucity of data available to demonstrate the range of histologic presentations of this entity, which makes it difficult to know what features are characteristic enough to warrant further work-up.

In this study, we present a series of 22 cases with histologically proven TVF and provide a comprehensive analysis of their histologic findings as well as their associated clinical and laboratory data, when available, to help provide a more holistic view of the range of histologic presentations seen in TVFs as well as the settings in which they are most commonly seen.

## Methods

### Case Identification, Report Itemization, and Clinical Review

Cases were identified using natural language search of pathology reports within the University of California-Los Angeles health electronic medical records. Reports referencing TVFs, tubulovenous herniation, or intravascular THP were identified, and the contents of the report and patient chart were analyzed for relevant clinical, laboratory, and histologic features. In total, 17 cases had accompanying laboratory data regarding hematuria status, and 16 had laboratory data regarding proteinuria. Collection of patient data for this study has been approved by the Office of the Human Research Protection Program (OHRPP) (IRB#20-001734).

### Histopathologic Examination

Of the cases identified, 19 were available for histopathologic examination either via digital whole slide image archive (n = 13) or physical glass slide review (n = 6). Cases were reviewed by a medical renal pathologist to identify pertinent histopathologic patterns. Representative photomicrographs of pertinent findings were taken using CellSens (Olympus Corporation, Tokyo, Japan) for glass slides and Aperio ImageScope (Leica Biosystems, Nussloch, Germany) for digital slides.

### Data Analysis

Data were organized and analyzed using Microsoft Excel (Microsoft Corporation, Redmond, Washington).

### Literature Review

Literature was identified using iterative keyword searches for the terms “tubulovenous communication,” “tubulovenous herniation,” “Tamm-Horfall polyp,” and “tubulovenous fistula” on PubMed and Google Scholar. The literature cited in each of the articles was also provisionally reviewed for relevance. All identified articles were reviewed for pertinent data.

## Results

A summary of all cases in this study is provided in Table 1. Twenty-one (95.5%) of the specimens were biopsies, and one (4.5%) was a resection. The most common indication for biopsy was acute kidney injury (n = 16, 72.7%). The patient population in the case series consisted of 15 males (87.2%) and 7 females (31.8%) according to the information recorded in the medical record as sex assigned at birth. The median age at time of biopsy was 66 years of age with ages ranging from 25 to 85 years. Sixteen (72.7%) of the cases were from native kidneys, whereas (27.3%) originated from renal allografts. Seventeen cases had accompanying urinalysis data. Of the 17 cases with data available regarding hematuria, 82.4% (n = 14) had microscopic hematuria, and 17.6% (n = 3) presented with gross hematuria. Proteinuria was also seen in 68.8% (n = 11) of the cases with available urinalysis data (n = 16).

**Table 1:** Summary of Cases

Case No.	Sex	Age	Presenting Symptoms	Primary Diagnosis	Additional Findings
1	M	41	AKI on CKD with MH	ATN	Mild diabetic glomerulo-sclerosis, moderate to severe arteriolar nephrosclerosis, and mild interstitial inflammation and fibrosis
2	M	44	Rejection monitoring	Mild ATI	
3	F	84	Nephrectomy for renal pelvis mass, MH	High-grade urothelial carcinoma and obstructive uropathy	
4	M	48	AKI after transplant, MH	ACR 1A	ATN/ATI
5	F	49	AKI after transplant, MH	ATN with focal neutrophil cast	Probably AMR
6	F	69	Lupus with CKD, MH	Diabetic glomerulosclerosis	ATN/ATI; patchy chronic active tubulointerstitial nephritis
7	M	41	AKI	ATI with calcium phosphate and myoglobin cast nephropathy	AIN with eosinophils
8	F	27	AKI, rhabdomyolysis, MH	ATI with myoglobin cast nephropathy and osmotic tubulopathy	
9	M	77	AKI	Light chain cast nephropathy, kappa light chain type	
10	F	68	Worsening CKD, MH	Severe arterial and arteriolar sclerosis with secondary FSGS exhibiting collapsing feature	ATN, chronic uric acid nephropathy
11	M	68	Worsening CKD, MH	Oxalate nephropathy	Secondary FSGS
12	M	72	AKI	Endocarditis-related GN and anticoagulation nephropathy	CIN, amyloid, DN, ATN
13	M	64	AKI on CKD, MH, GH	Chronic active tubulointerstitial nephritis	ATN and DN
14	M	69	AKI after transplant	ACR 1B	ATN, early DN
15	M	25	AKI, rhabdomyolysis	ATN with myoglobin casts	
16	M	77	AKI, GH, MH	Mild ATI and prominent TVF	
17	M	30	AKI, MH	Lupus nephritis, class III	
18	M	43	AKI after transplant	ACR and focal hemorrhagic infarction	
19	M	46	Delayed graft function	ATN with focal neutrophil cast	
20	F	84	AKI, MH	ATN, AIN, and neutrophil casts	
21	F	68	AKI after antibiotics, MH	AIN	
22	M	72	AKI, GH	AIN and vasculitis	ATN, minimal MN

Abbreviations: ACR, acute cellular rejection; AIN, acute interstitial nephritis; AKI, acute kidney injury; AMR, antibody-mediated rejection; ATI, acute tubular injury; ATN, acute tubular necrosis; CIN, chronic interstitial nephritis; CKD, chronic kidney disease; DN, diabetic nephropathy; FSGS, focal and segmental glomerulosclerosis; GH, gross hematuria; GN, glomerulonephritis; MH, microscopic hematuria; MN, membranous nephritis.

Histologically, nearly all cases (n = 21, 95.5%) exhibited acute tubular injury or necrosis (Table 2). Interstitial nephritis was observed in 9 (56.3%) of the native kidney cases. Rejection was observed in 4 (66.7%) of the transplant cases with 3 (50.0%) exhibiting acute cellular rejection and 1 (16.7%) exhibiting antibody-mediated rejection. Interstitial fibrosis and tubular atrophy were also observed in a majority (n = 13, 59.1%) of cases with a higher proportion (n = 12, 75.0%) being observed in native cases when compared with transplant (n = 1, 16.7%). Glomerular disease (including diabetic glomerulosclerosis, focal segmental glomerulosclerosis, membranoproliferative glomerulonephritis, lupus nephritis, and immune complex-mediated glomerulonephritis) and obstructive features (characterized by THP reflux) were seen in just under half of all cases (n = 10, 45.5% for both). A majority (n = 16, 72.7%) of cases exhibited intraluminal casts of any type with neutrophilic casts being observed more frequently (n = 8, 36.4%). Other casts observed in this series included Tamm-Horsfall, calcium crystal, myoglobin, red blood cell, and light chain casts.

**Table 2:** Histopathologic Findings in Cases Examined

Sample Characteristics	Total No. (%)	Native No. (%)	Transplant No. (%)
	N = 22	N = 16	N = 6
Acute tubular injury/necrosis	21 (95.5)	15 (93.8)	6 (100)
Interstitial nephritis	-	9 (56.3)	-
Interstitial fibrosis and tubular atrophy	13 (59.1)	12 (75.0)	1 (16.7)
Mild	6 (27.3)	6 (37.5)	0
Moderate	5 (22.7)	5 (31.3)	0
Severe	2 (9.1)	1 (6.3)	1 (16.7)
Glomerular disease	11 (50.0)	10 (62.5)	1 (16.7)
Obstructive features	10 (45.5)	7 (43.8)	3 (50.0)
Casts (any type)	16 (72.7)	13 (81.3)	3 (50.0)
Neutrophilic	8 (36.4)	6 (37.5)	2 (33.3)
Tamm-Horsfall	4 (18.2)	3 (18.8)	1 (16.7)
Calcium crystal	2 (9.1)	2 (12.5)	0
Myoglobin	2 (9.1)	2 (12.5)	0
Red blood cell	1 (4.5)	1 (6.2)	0
Light chain	1 (4.5)	1 (6.2)	0
Arteriolar hyalinosis/arteriosclerosis	13 (59.1)	13 (81.3)	0
Rejection	-	-	4 (66.7)
Acute cellular rejection	-	-	3 (50.0)
Antibody-mediated rejection	-	-	1 (16.7)

**Figure 2:**
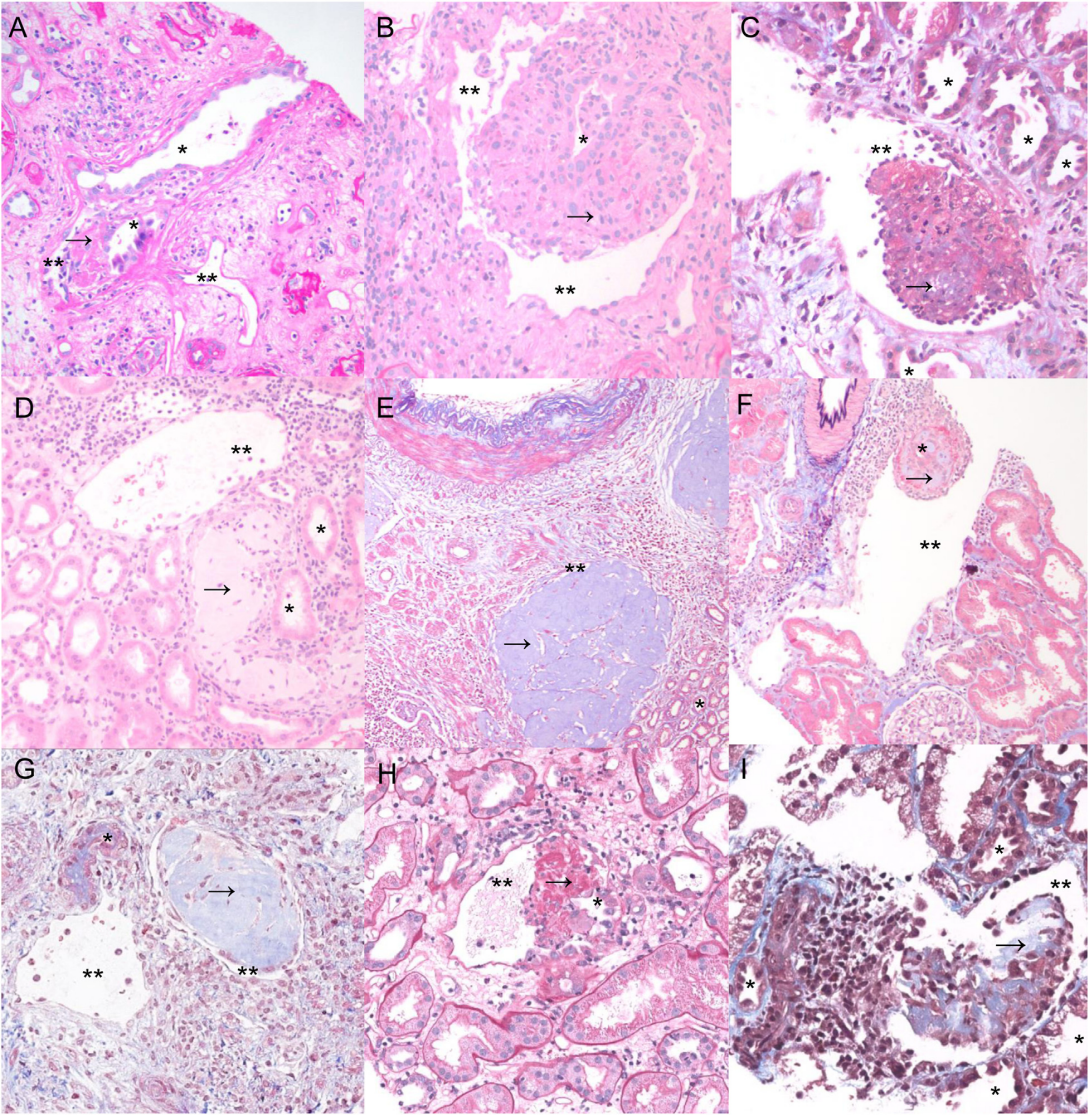
Histopathologic diversity seen in tubulovenous fistulas. Series of histologic photomicrographs depicting the range of presentations seen in cases with evidence of TVF. When relevant, the tubule (∗), vein (∗∗), and THP (arrow) are demonstrated. Key histologic presentations include THP with associated fibrin (A, B, C, F, H, I), THP in a vein adjacent to a tubule (C, D, G), herniation of the tubule and THP into the vein (B, F), massive THP deposition in large renal veins (E), and observed fistula formation between the tubule and vein (A, H). Abbreviations: TVF, tubulovenous fistula; THP, Tamm-Horsfall protein.

Histopathologic examination of the cases in this series showed a spectrum of histologic presentations (Figs 1 and 2). Histologic findings associated with the TVF included THP localization, inflammation, and alterations to tubular or venous structure. Inflammation was commonly focally increased proximal to the TVF. The cellular reactions associated with TVF were most commonly lymphocytic, but acute, lymphoplasmacytic, and granulomatous reactions were also observed. Localization of THP was seen in proximal tubules (reflux), within the lumen of a vein, and in the interstitium. The morphology of THP aggregates tended to either be purely nodular or polypoid with no admixture of other obscuring elements or exhibited abundant admixed fibrin with or without associated inflammation and tubular epithelial cells. Tubular or venous structural aberrancies included herniation of the tubule into the vein as well as a clearly defined fistula between the tubule and vein. Notably, the physical connection between the tubules and vein was not able to be observed in all cases and inferred based on the presence of THP in the lumen of veins associated with cellular reaction and fibrin accumulation. Additionally, most cases exhibited variable amount of intratubular blood. The venous involvement in most cases was singular and involving arcuate-sized veins. Immunohistochemical characterization of the vascular space on select cases showed the vascular spaces were lined by endothelial cells (Fig S1).

Based on our review of the literature, there have been 9 reports of this phenomenon (Table 3).^1^, ^2^, ^3^, ^4^^,^^7^, ^8^, ^9^, ^10^, ^11^ The first reference of a connection, communication, or herniation between the renal tubule and vein was reported in 1941 by Dunn.^1^ This served as the first limited case series about this phenomenon focused on its appearance in patients with crush injuries. Decades later, a larger case series was performed by Barrie in which this histologic finding was searched for in 5,000 autopsies.^3^ This report described 68 cases of a potential herniation of the tubule with urinary proteins into either the lymphatic spaces or the arcuate vein. These cases were more commonly associated with obstructive uropathy/reflux features; however, in cases without reflux, severe tubular injury was frequently identified. Although this study looked at a large number of cases, it is unclear what criteria was used for identifying these cases, and the numbers of cases from each type (lymphatic or venous) were not delineated. The first report characterizing the biochemical makeup of these polyps was by Solez and Heptinstall in 1978, in which immunofluorescence detected strong THP positivity in the polyps.^2^

**Table 3:** Review of the Literature

Paper Authors	Year	No. of Cases	Age	Clinical Setting	Histologic Associations	Reference
Lilley and Zuckerman	2025	22	25-85 y	Varied: incidental findings in medical renal cases for AKI, transplant monitoring, or neoplastic nephrectomy	Intravenous THP with or without fibrin/mixed inflammation, focally increased inflammation, herniation of tubule into vein or discrete TVF. Associated with acute tubular necrosis (95.5%), casts (72.7%), and IFTA (59.1%).	This study
Almosa et al^11^	2015	1	30 y	Hematuria and flank pain	Massive tubular hemorrhage, IFTA, and interstitial/intravenous THP	^11^
Higgins et al^10^	2006	1	57 y	Acute gross hematuria, flank pain, and hematoma	Intravenous THP with a variable amount of fibrin, interstitial inflammation, intratubular hemorrhage	^10^
Billis et al^9^	1990	1	24 y	Asymptomatic gross hematuria with diminished renal vein blood flow	Massive intratubular hemorrhage, THP deposition in the corticomedullary junction herniating into thin-walled veins. Some polyps were covered by fibrin.	^9^
Kawaguchi et al^7^	1989	14/20	39-80 y	Autopsies of patients with MM	Polypoid projection of THP into the vein was seen in 70% of cases. TVF was seen in 20% of cases.	^7^
Cohen et al^4^	1984	2/13	NA	Transplant allografts	No tubular damage identified. The 2 cases with intravascular THP localized the material to the peritubular capillaries. No reflux features noted in this series.	^4^
Solez and Heptinstall^2^	1978	6^a^	2 y	Horseshoe kidney with hydronephrosis	Demonstration of THP by IF. THP polyps in the interstitium, tubules, and arcuate veins. Some THP polyps with associated inflammatory response or fibrin. THP polyps were rarely occlusive. Reflux noted.	^2^
			8 m	Bilateral vesicoureteral reflux		
			45 y	Kidney allograft with delayed graft function and rejection		
Iliff and Galdabini^8^	1972	3	18 y	Flank pain and gross unilateral hematuria	Intratubular hemorrhage, THP in arcuate vein lumina, tubular damage, venous thrombi, edema	^8^
			23 y	Heavy unilateral bleeding after biopsy		
			15 y	Flank pain and gross unilateral hematuria		
Barrie^3^	1961	68^b^	NA	Autopsies of patients with intravenous or lymphatic herniations	Tubulovenous thromboses (also called extrusion polypi), urinary glycoproteins herniating into the venous space. Arcuate vein involvement. Some cases had associated fibrin. Herniations were more commonly observed in patients with reflux (48/68). In the cases without features of reflux, severe tubular injury was noted (20/68).	^3^
Dunn^1^	1941	2	18 y, 51 y	Patients with crush injuries	Cast material in tubules, distension of tubules, rupture of tubular epithelial and herniation into adjacent veins.	^1^

Abbreviations: AKI, acute kidney injury; IF, immunofluorescence; IFTA, interstitial fibrosis and tubular atrophy; mo, months of age; MM, multiple myeloma; NA, not applicable; THP, Tamm-Horsfall protein; TVF, tubulovenous fistula; y, years of age.

a Three of the patients from this article were reported previously by Iliff and Galdabini^8^ in 1972.

b This article describes 2 different histologic entities, those with herniation of urinary material into lymphatics (lymphogenous polypi) and those with herniation from a tubule to a vein (extrusion polypi). It is unclear how many of the cases described in this report refer to the specific tubulovenous connection explored in this article.

Since its initial characterization, multiple cases of massive intratubular hemorrhage were described.^8^, ^9^, ^10^, ^11^ These cases tended to have a unique clinical presentation and typically required nephrectomy. Across the reported literature, several disease states were noted to be correlated with the formation of TVFs including crush injuries, obstructive uropathy, multiple myeloma, transplant rejection, or medical instrumentation. However, an association or cause was not able to be determined in some cases.^9^ Nearly all cases described involvement in arcuate-sized veins, although reports of lymphatic and peritubular capillary involvement were also seen. Other histologic findings commonly reported included characterization of the THP polyps with or without mixed inflammation and fibrin. Other associated renal cortical changes were also noted including rejection (in allograft cases), interstitial fibrosis or tubular atrophy, tubular casts (including hemoglobin, myoglobin, and protein casts), tubular damage, as well as focally increased inflammation/edema.

## Discussion

TVFs are an uncommon finding on histologic examination of kidney tissue. Here, we present the largest case series to date exploring the clinical, laboratory, and histopathologic diversity seen in cases with at least one microscopically evident TVF. Importantly, this study sheds light on the diversity that can be seen in this pathologic entity highlighting the importance of increased awareness of this entity and further characterization of its associated pathologies, clinical presentations, and prognosis or natural history.

Although the finding is often incidental, its presence could potentially explain hematuria in patients with no evidence of glomerulonephritis or other histopathologic explanation for hematuria. No prior research has been able to determine a potential pathophysiological explanation for the formation of TVFs. However, in this study, a vast majority of the cases with TVFs also had concurrent acute tubular necrosis because of ischemia, casts, or tubulointerstitial nephritis. It is likely these tubular injuries result in tubular rupture which when occurring in close proximity to a vein could result in disruption of the vein wall and microscopic communication between the tubular and venular lumina. Because acute tubular injury, in the absence of glomerular disease, because of ischemia, intrinsic or extrinsic toxins, or tubulointerstitial nephritis is not typically associated with development of hematuria, the presence of TVFs in this clinical setting could explain gross or microscopic hematuria.

The differential diagnosis for these findings could include renal arcuate vein thrombosis.^16^^,^^17^ Although these entities can exhibit histologic and pathophysiologic overlap, the occlusive substance in a TVF is THP which has a distinct histologic appearance. However, in cases involving admixed fibrin and/or mixed inflammatory cells, this distinction can be challenging and could require examination of multiple sections or immunohistochemical staining for THP. In cases in which renal arcuate vein thrombosis cannot be fully excluded, it is important to thoroughly examine multiple histologic levels and look for other associated histopathologic findings we describe in this case series.

One of the challenges in the detection of TVFs on core needle biopsy sampling is the probability sampling such a focal lesion. Further, it is not fully understood how diffuse these lesions tend to be when they occur and what percentage of the renal parenchyma needs to be affected by TVFs to cause gross/microscopic hematuria. Taken together, it is difficult to determine how often TVFs are detected when present. Focality and sampling challenges further cloud our understanding of this entity as well as our estimation of how common or uncommon this entity is in diverse clinical settings.

Limitations to this study include its retrospective nature and the absence of full laboratory data, clinical follow-up, and original slides in some cases. Because of this, there are still unanswered questions regarding outcomes in these patients, including if there is an altered healing response to interstitial inflammation because of the presence of THP. Additionally, uromodulin immunostaining is not performed in our laboratory limiting immunohistochemical confirmation of the protein. Furthermore, because of the under-recognition of this finding it is likely that many cases at our institution were not discovered because of the absence of note within pathology reports.

This study offers a unique, quantitative account of the common associated clinical, laboratory, and histopathologic features seen in cases with histologically evident TVFs. Importantly, this study identifies acute tubular necrosis and tubular injury as a potential risk factor in the development of TVFs. Interstitial nephritis, and intratubular casts could represent additional pathologic findings that could mechanistically lead to TVF formation. Prior research in the transplant setting has demonstrated tubular basement membrane attenuation or discontinuity in the setting of rejection,^18^ which, if similar effects to the basement membrane are seen outside of the transplant setting, could partially explain the formation of TVFs in association with tubular injury and interstitial inflammation. Further, hematuria is rare in patients with acute tubular necrosis.^5^ Thus, in patients with only findings of tubular injury with no other potential explanatory clinical or pathologic findings, focal TVF formation could be a potential mechanism for the development of hematuria in these patients.

This study also provides a comprehensive account of the common histopathologic manifestation of this condition including focally increased interstitial inflammation, tubule rupture, intravenous or interstitial THP, and definitive demonstration of a TVF. Importantly, this study shows that examination of multiple levels of tissue may be required to clarify the diagnosis and not every case will have a perfectly demonstrated pathologic connection. Moving forward, it is important to continue studying this entity and encouraging thorough clinical and pathologic examination of identified patients, so we can better understand its clinical relevance and long-term prognosis.
