# Supplementary material for: Microscopic Tubulovenous Communications in Nonneoplastic Kidneys: A Single-Center Case Series and Review of the Literature
Source: Kidney Med. 2025 Jul 16;7(9):101071. doi: 10.1016/j.xkme.2025.101071 (PMC12396424; doi:10.1016/j.xkme.2025.101071)
Supplement: Supplementary File (PDF) — Figure S1. [file mmc1.pdf]

Figure S1: Immunohistochemical Characterization of Tubulovenous Fistulae

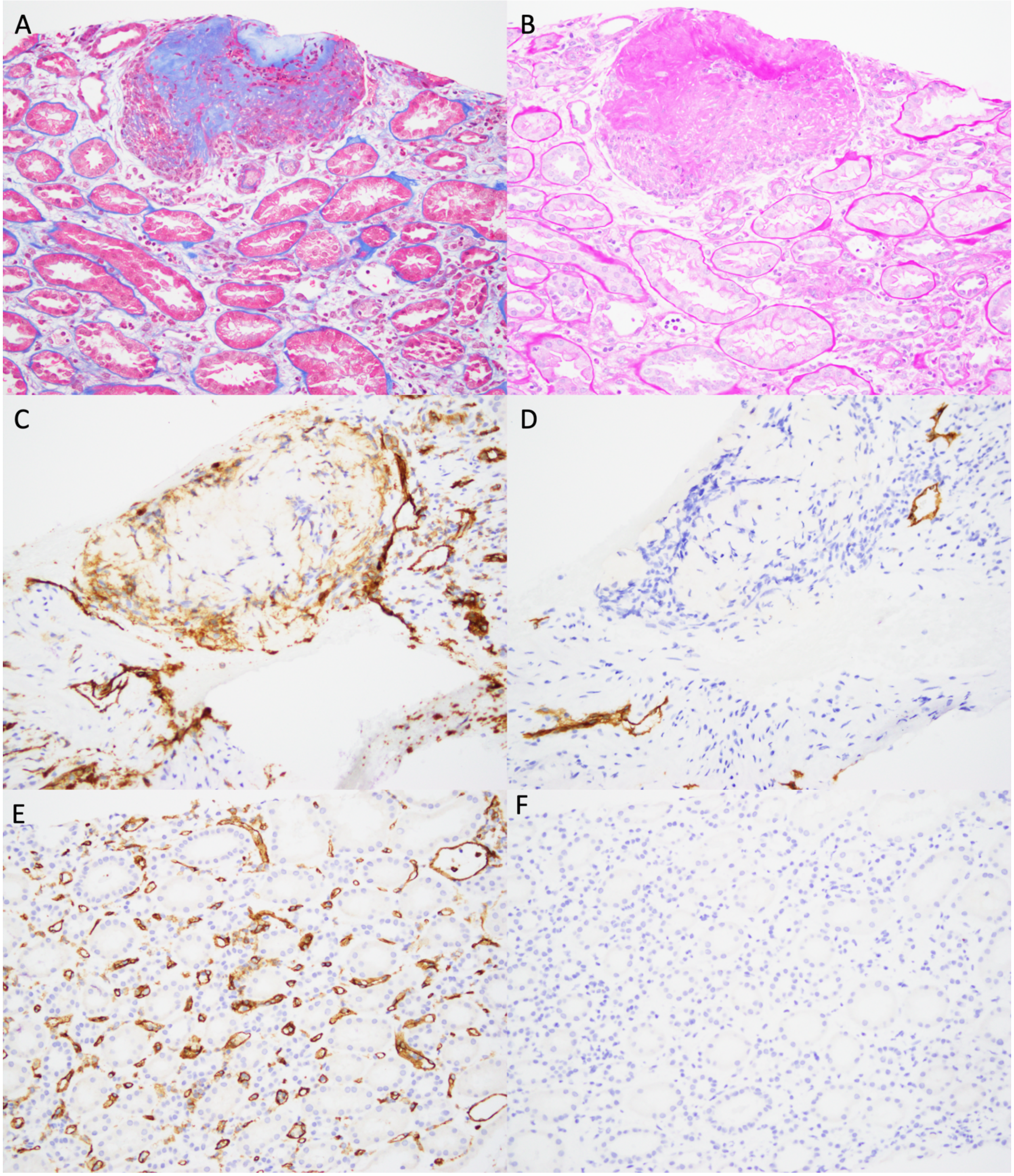

Series of photomicrographs depicting a tubulovenous fistula (A-D) stained with Masson's trichrome (A), periodic acid-Schiff stain (B), CD31 (C), and D2-40 (D). Additional photomicrographs depicting adjacent, uninvolved kidney parenchyma with

numerous vessels located between the tubules, including dilated veins with attenuated vascular walls staining with CD31 and minimal lymphatic vessels staining with D2-40.
